# Effect of Conductive Carbon Black on Mechanical Properties of Aqueous Polymer Binders for Secondary Battery Electrode

**DOI:** 10.3390/polym11091500

**Published:** 2019-09-14

**Authors:** Hongjiu Hu, Bao Tao, Yaolong He, Sihao Zhou

**Affiliations:** 1 Shanghai Institute of Applied Mathematics and Mechanics, School of Mechanics and Engineering Science, Shanghai University, Shanghai 200072, China; 2 Shanghai Key Laboratory of Mechanics in Energy Engineering, Shanghai 200072, China

**Keywords:** aqueous polymer binder, conductive carbon black, mechanical properties, adhesive performance, microstructure

## Abstract

To predict the cyclic stability of secondary battery electrodes, the mechanical behaviors of polymer binders and conductive composites (BCC) is of great significance. In terms of uniaxial tension, tensile stress relaxation, and bonding strength tests, the present study encompasses a systematic investigation of the mechanical properties of two typical aqueous binders with different contents of Super-S carbon black (SS) under a liquid electrolyte. Meanwhile, the microstructure of cured film and the surface morphology of the bonding interface are investigated in detail. When the weight ratio of SS increases from 0% to 50%, the cured BCC films manifest a higher ratio of tensile strength to modulus and a shorter characteristic relaxation time. Moreover, suitable loadings of SS can improve the tensile shear strength and remarkably reduce the percentage of interface failure of aqueous polymer-bonded Cu current collector. Nevertheless, an excess of carbon black amount cannot maintain its enhancing effect and can even impair the adhesive layer. Finally, a sodium alginate-based polymer composite holds much more superior mechanical properties than the mixture of sodium carboxymethyl cellulose and styrene-butadiene rubber at the same content of carbon black. Noticeably, the two kinds of aqueous polymer doped by 50 wt % of SS exhibit the best adhesive properties.

## 1. Introduction

A polymeric binder that is applied as an inactive in the composite anode and composite cathode for lithium secondary battery is only 1–10 wt % loading of active particles [1,2,3]. However, a polymeric binder is an essential component for keeping the mechanical stability and electrical integrity of an electrode structure. Compared to the popular poly(vinylidene difluoride) (PVdF) binder that contains toxic organic solvent, aqueous polymer adhesives have prominent advantages such as high bonding strength, good electrochemical properties, relative cheapness, and environmental friendliness. Therefore, sodium carboxymethyl cellulose (CMC), styrene-butadiene rubber (SBR), polyacrylic acid (PAA) [4,5], polyvinyl alcohol (PVA) [6], and sodium alginate (SA) [7,8,9], among others that use water as a solution, have been the potential choices for the electrodes in advanced lithium secondary batteries. Among the investigations of advanced electrodes based on water soluble polymer adhesives, considerable efforts have been made to reveal the effect of binder nature on the reversibility and cyclability of the cell. It is found that the mixtures of CMC and SBR reveal the higher coating adhesion contrast to PVdF, which leads to an obvious enhancement in the cycle stability and rate performance of metal oxide electrodes [10,11,12], graphite [13], silicon [14] anodes, and sulfur cathode [15]. Due to a great deal of carboxyl groups, SA can boost the adhesion strength of high-capacity anodes on the copper current collect, thus improving the cycle life of secondary batteries [7,8,9]. For example, activated carbon/SA electrodes can sustain up to 2000 charge/discharge cycles in electrolytes that contain Li-salt [8]. Similarly, PAA has strong hydrogen bonds with active particles and the current collector. As a result, electrodes with a PAA binder exhibited good cyclic performance [16]. Further, it has been identified that the cohesion properties of polymer binders have played a pivotal role on the electrode integrity and electrochemical stability during lithiation/delithiation. Owing to high tensile strength and stiffness of the cured binder film, a Mo_6_S_8_ cathode with PAA and PVA exhibited good mechanical stability, capacity retentions, and rate capabilities for rechargeable magnesium batteries [17]. Li et al. also reported the influence of binders elastic modulus on bending deformation and Vegard stress in Si composite electrodes with SA, Nafion, and PVdF [18].

It is well recognized that composite electrodes are generally composed of an active particle, polymer binder, and conductive carbon black (CB). As the specific surface area of CB is far larger than that of the active material, the electrode system can be regarded as an active particle being bound together by a CB filled polymer composite [19], which electrical and mechanical properties naturally impact the electrode performance. Hence, the effect of conducive additive on polymer binder has been gradually attracting scientific and industrial interest [19,20,21,22,23,24]. Generally, increasing the conductive agent contributes to a higher electronic conductivity of the polymer composites. However, Liu investigated the impedance of PVdF evolved with the different content of acetylene black (AB) and observed that the DC conductivity increased and plateau at AB/PVdF = 0.2 (weight ratio) [20] for the electrode with a high loading of active material; the lower the AB/PVdF ratio, the better the cell power performance [20,21]. In order to uncover the effect mechanism of AB on the electrode properties, Takahashi et al. tested the stress-strain behavior of the conductive agent filled polymer binder composite (AB/PVdF = 0.2), which was immersed in an electrolyte solution. Further, the maximum stress and Young’s modulus of PVdF were found to be weakened when adding carbon black [23]. Similar effects of conductive carbon black on the mechanical properties of PVdF were obtained by Zheng et al. [21] and Grillet′s group [24]. However, as the content of Super-S carbon black increased, it enhanced poly(vinylidene fluoride-co-hexafluoropropylene)-based composites. Hence, the CB appears to act upon a complicated mechanical role when added into the fluorinated elastomer. It is still not clear how the conducting agent impacts the mechanical behavior of the electrode binders, especially for an aqueous polymer adhesive system. The knowledge of adhesive and cohesive properties of the water-soluble binder with various carbon black loadings under electrolyte solution conditions can not only allow for a better understanding of the mechanical behavior and failure mechanism of electrode structures but the basis of aqueous adhesive design and application for high- performance secondary batteries. Up until now, no previous studies have paid close attention to this issue.

In this study, we focus on exploring the influence of conductive carbon black on mechanical properties of aqueous polymer binders under realistic condition. Herein, we aim to provide a simple and accurate basis for theoretical modeling and applications with high-energy density electrodes. The typical water-soluble polymers (SA and CMC/SBR) were filled with CB at different loadings when preparing electrode binders. Following that, a series of uniaxial tension and stress relaxation were carried out to identify the quasi-static mechanical behavior of cured adhesive films subjected to organic electrolytes, the tensile strength, Young′s modulus, and characteristic retardation time. Furthermore, the bonding strength of the adhesives with varying concentrations of carbon black were compared according to ASTM D3165. In order to clarify the effect mechanism of inorganic nanoparticles on the mechanical response of the polymer binder composite, scanning electron microscopy (SEM) and optical microscopy were used to check the change in the microstructure of cured film and the failure interface between the binder and current collector, respectively.

## 2. Experimental

### 2.1. Materials and Samples

The mixtures of CMC (CMC 2200, Daicel, Japan) and SBR (BM450, Zeon, Japan) with a 1:1 weight ratio, as well as SA (Alladin, Shanghai, China), were separately adopted as the matrices of the binder and conductive composites (BCC). Moreover, Super-S carbon black (SS, CM65, Kejing, Shenzhen, China) had a weight ratio of 0%, 20%, 35%, 50%, and 60% and used as the dispersed phases in BCC, respectively. SA aqueous solutions were prepared by dissolving polymer powder in distilled water at 80 °C for 3 h under stirring at 90 r/min. SS with an average particles size of 40 nm were added into the solution which was cooled at 50 °C, and thoroughly mixed under mechanical and ultrasound agitation. Meanwhile, SS-CMC/SBR solutions were obtained via a similar procedure. Subsequently, the samples were prepared as follows:

(1) Cured BCC films: after being degassed for 30 s, the SS-polymer mixed aqueous solutions were spread on Teflon-coated glass plates and dried at 25 ± 2 °C and 50% relative humidity (RH) for seven days. Then, the obtained films with thickness of 100 μm were cut into rectangular samples (5 mm × 20 mm) for uniaxial tension and stress relaxation and preserved in a desiccator with recently dried silica gel as samples for use.

(2) BCC bonded current collector specimen: Cu foil used in this experiment had dimensions of 100.0 mm (L) × 25.4 mm (W) × 1.7 mm (T) with an adhesive area (S) of 25.4 × 12.5 mm^2^. The adherend surfaces cleaned with acetone were bonded for single-lap-joint laminated assemblies and the thickness of the BCC adhesives were about 0.2 mm.

### 2.2. Test Methods

The samples were immersed in the liquid electrolyte with 1.1 M LiPF_6_ in a mixed solution of EC and DMC at 1:1 volume ratio (LiPF_6_-EC/DMC) for 48 h under room temperature before the mechanical experiments.

(1) In order to obtain the mechanical behavior of cured BCC films with the electrolyte solution, a quasi-static tension experiment was carried out in the strain rate of 5 × 10^−3^·s^−1^ using a DMA (TA Q800, TA Instruments-Waters LLC, New Castle, DE, USA) with a submersion film/fiber clamp.

(2) With the view of elucidating the relaxation response of cured BCC films, tensile stress relaxation experiments were fulfilled at 1.0% strain, which was chosen from the linear section of the stress-strain curve of the polymer composites. The stress required to maintain constant strain was recorded during about 60 min in uniaxial tension, with a sampling rate of 10 Hz.

(3) For the purpose of clarifying the effect mechanism of the carbon black on the mechanical properties of the aqueous polymer binder, the surface morphology of cured BCC films with different SS contents were imaged using SEM (Gemini SEM-300, Carl Zeiss, Jena, Germany).

(4) The bonding properties of BCC were assessed according to ASTM D3165. The tensile shear test for BCC glued Cu foil was performed at a crosshead speed of 1.27 mm/min by a universal test machine (Zwick-Z020, Ulm, Germany) under ambient temperature. Following this, an optical microscope (Zeiss Smart zoom 5, Carl Zeiss, Jena, Germany) was used to check the surface morphology of the failure interface between the binder and current collector. The obtained optical microscopy images were then transformed into binary images using a MATLAB program based on the region growing method.

## 3. Results and Discussion

### 3.1. Uniaxial Tensile Properties of Cured BCC Film

**Figure 1:**
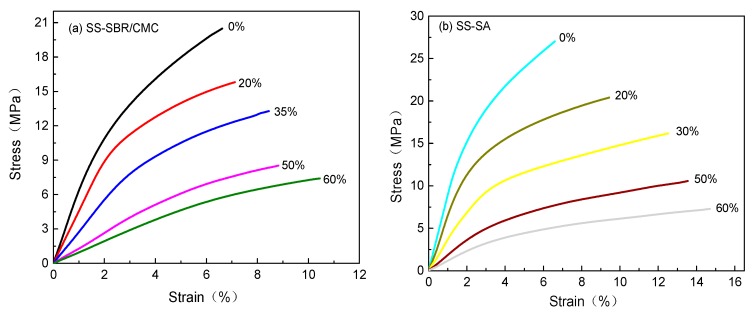
Effects of carbon black (SS) content on the tensile stress-strain curves of cured conductive composites (BCC) films.

**Figure 2:**
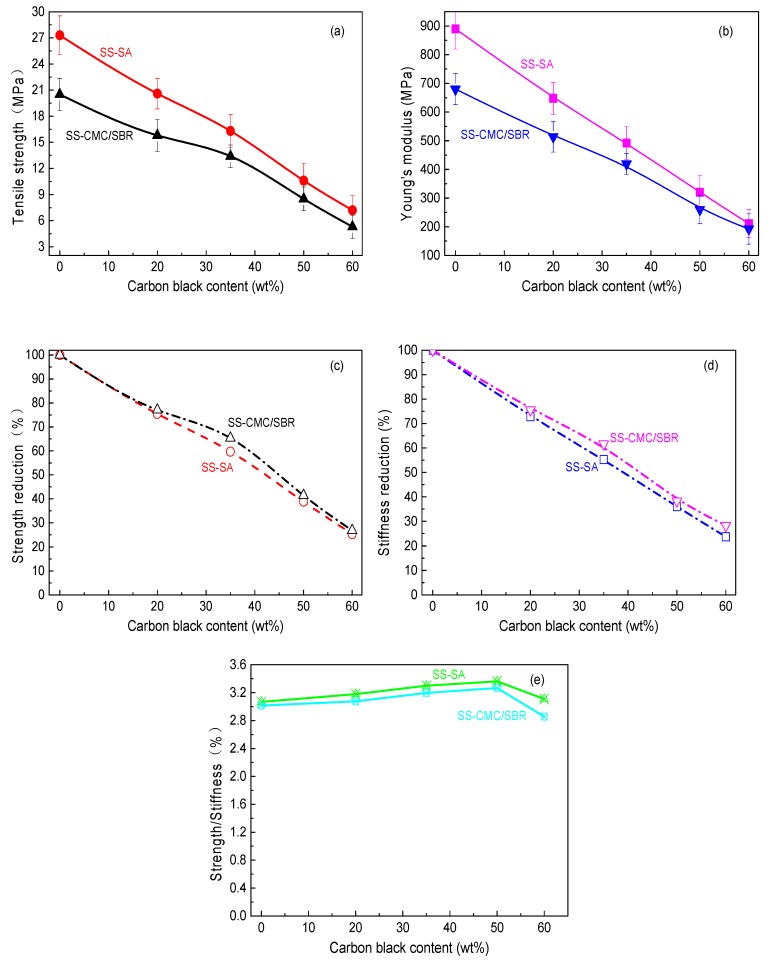
Evolution of (**a**) maximum stress; (**b**) Young′s modulus; (**c**) strength reduction; (**d**) stiffness reduction and (**e**) ratio of strength to stiffness of BCC films with SS content for different aqueous polymer matrices.

The uniaxial tensile deformation of cured BCC in 1.1 M LiPF_6_-EC/DMC is shown in Figure 1. For SBR/CMC or for SA films as seen Figure 1a,b, respectively, the engineering stress-strain curves of testing samples without SS carbon black nearly exhibited a characteristic behavior of the brittle polymers, which broke after yielding without a cold drawing. Due to the addition of SS carbon black, the stiffness decreased, while the strain after yielding increased, resulting in approach to a fractured response of the ductile polymer. Meanwhile, it was observed that the tensile mechanical properties of BCC film samples were strongly dependent on the aqueous polymer type and conductive agent loading. Furthermore, the variation of the maximum stress and Young′s modulus determined from the stress–strain curves with carbon black content are plotted in Figure 2.

As seen in Figure 2a,b, SA and its composite revealed better resistance to tensile rupture than SBR/CMC matrices with the same SS content. When the loadings of carbon black increased, tensile strength and stiffness decreased remarkably for the cured BCC film. It can be clearly observed from Figure 2c,d that the composite samples doped with 60% SS had about 25% maximum stress (σ*_b_*) and Young′s modulus (*E*) of their unfilled counterparts. These results are similar to the evidence that conductive acetylene black filled PVdF composites showed a lower strength and stiffness [21,23,24]. On the contrary, the evolution of σ*_b_* and *E* contrasted with the ratio (σ*_b_*/*E*) of BCC films rose as the loading of SS increased from 0% to 50%, as shown in Figure 2e. However, it gradually decreased with the further increase of carbon black. Moreover, with an increasing concentration of SS particles, BCC films exhibited higher elongation (ε*_f_*) at a fracture. Therefore, adding the suitable loading of conductive carbon black (≤50% SS) can help the aqueous polymer improve its resistance to tensile rupture due to an increased σ*_b_*/*E* and ε*_f_* of the elastomers. 

It should be noted that neat SA films revealed better tensile properties compared to CMC/SBR; the effect of the conductive agent addition on the reduction of σ*_b_* and *E* is relatively noticeable for SA matrices. Nevertheless, the ratio of the tensile strength to modulus and the breaking elongation of SS-SA is larger than SS-CMC/SBR at the same carbon black content from 0% to 60%. This indicates that any SA-based composite may hold great promise for application in the field of secondary battery electrodes.

### 3.2. Tensile Stress Relaxation Behaviors of Cured BCC Film

**Figure 3:**
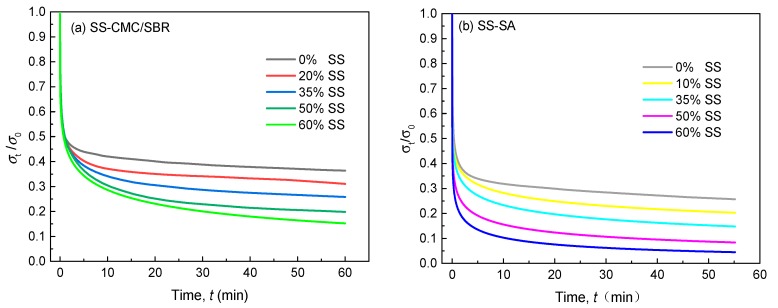
Normalized stress relaxation curves of cured (**a**) SS-SBR/CMC and (**b**) SS-SA films.

**Figure 4:**
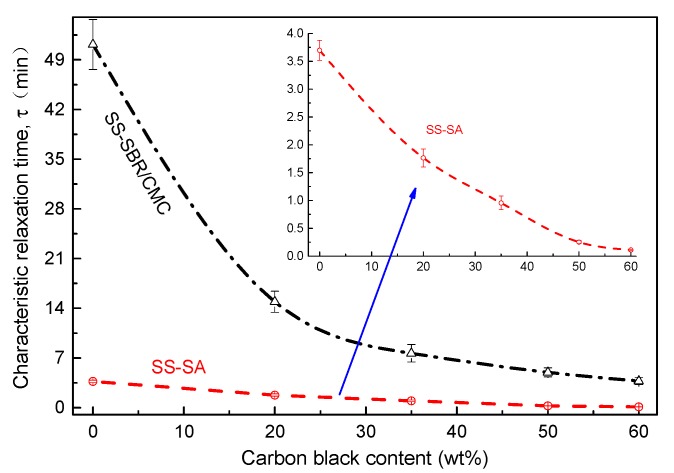
Evolution of the characteristic relaxation time of BCC films against carbon black content.

Under constant tensile strain, the variation of normalized stress versus time for conductive carbon black filled aqueous polymers in 1.1 M LiPF_6_-EC/DMC is shown in Figure 3a,b, respectively. All curves exhibited typical viscoelastic behavior (time-dependent stress reduction), but with different degrees of relaxation, which demonstrated that the polymer type and SS content had played a pivotal role in the stress relaxation process. We found that the stress level of SS-CMC/SBR steeply decreased up to about 40% of initial value (*t* = 0) after 5 min and the higher the loading of carbon black, the more rapid the decrease in stress. Similar stress relaxation behavior was observed for SA as the host polymer. The decays also got much more distinct with increasing SS concentration. This implies that the addition of conductive carbon black accelerated the relaxation process of aqueous polymers in the liquid electrolyte. In order to delve into the underlying mechanism, the Kohlrausch-Williams-Watts (KWW) model, as seen in Equation (1), was attempted to further analyze the decay in tensile stress—σ(*t*) of the aforementioned polymer composites. For all of the above tensile stress relaxation curves, the relaxation time (τ) was obtained using a 1stOpt^®^ non-liner regression software; its evolution against carbon black content are plotted in Figure 4. As the regression correlation coefficients are close to 1, the KWW time-decay formula can describe the stress relaxation behavior of aqueous polymers filled with SS carbon black.

(1) $$\frac{\sigma \left(t\right)}{\sigma_{0}}=\exp{\left(-\frac{t}{\tau }\right)}^{\beta }$$

where σ_0_ is the initial relaxation stress (*t* = 0), *t* is time, τ is characteristic relaxation time at which σ(*t*)decays to the value 1/e, and the exponent β describes the distribution in the limits of 0 < β <1.

As seen Figure 4, due to the relatively higher stiffness (elasticity) of the polymer chain, pure SA and SS-SA experienced a far shorter relaxation process beyond CMC/SBR and its composites. Moreover, the conductive carbon black significantly impacted the characteristic retardation time (τ), which was smaller in the doped composites than their undoped counterparts. Noticeably, the values of τ decreased up to over 90% as the SS concentration in the polymer composite rose from 0% to 60%. Therefore, the addition of the filler greatly enhanced and sped up the stress relaxation process in BCC films. This may be because SS particles were inserted into different polymer molecular chains. The electrostatic forces of these nano-fillers created a mutually exclusive system that forced molecular chains to become a looser framework, leaving extra free volume. Meanwhile, the plasticization effect of organic electrolyte exacerbated the speed of the slip movement of molecular chains, which facilitated the structure relaxation of BCC films.

### 3.3. Microstructure of Cured BCC Films

**Figure 5:**
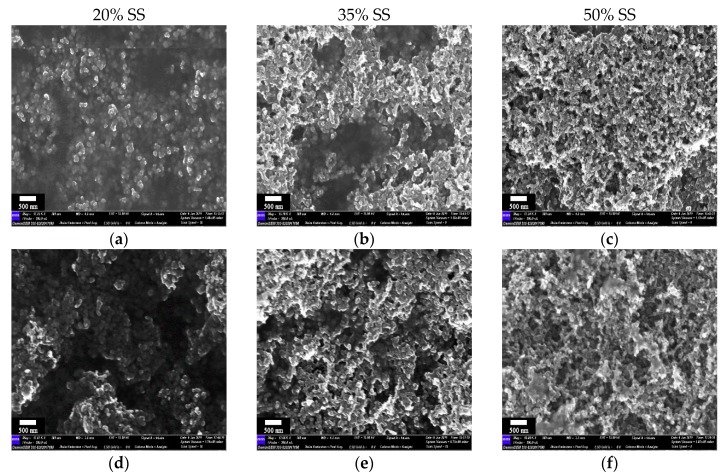
Effects of carbon black content on SEM of cured BCC films (SS-CMC/SBR: (**a**–**c**); SS-SA: (**d**–**f**)).

In order to disclose the effect mechanism of the carbon black on the aqueous polymer binder, we used further analysis according to the surface morphology of cured BCC film via SEM, as shown in Figure 5. At a low carbon black concentration (Figure 5a,d), SS particles were nonuniformly embedded into the continuous polymer phase. With increasing carbon black content (Figure 5b,c,e,f), the polymer island was reduced to much smaller domains. Meanwhile, SS aggregates became more visible on the surface with the retreat of the polymer host and these phenomena were comparatively obvious for SS-SA composites. At the 50% SS loading, the individual domains of CMC/SBR or SA were difficult to observe. Thus, the inhomogeneous distribution of carbon black particles may have induced a mass of open volume defects and free volume in the polymer composite, leading to a decrease in the tensile strength and stiffness of cured BCC films.

### 3.4. Bonding Performance of BCC Adhesive

**Figure 6:**
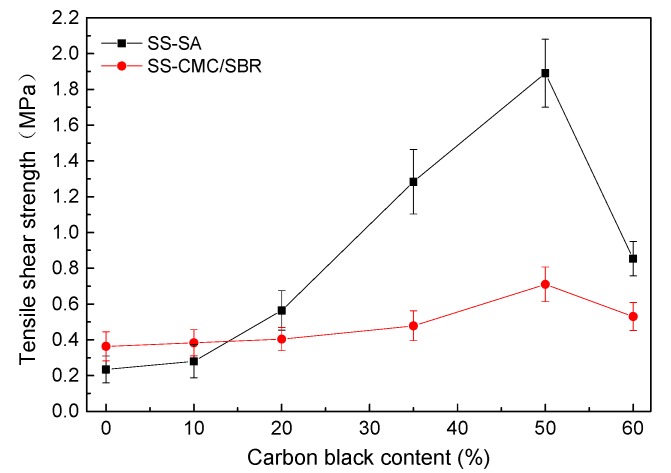
Effect of carbon black (SS) content on tensile shear strength of BCC with various aqueous polymers.

Besides the afore mentioned mechanical behavior of the cured polymer film, another key issue is the interface performance between the current collector surface and the bulk BCC adhesive. In order to evaluate the role of conductive carbon black on the bonding properties of water-based polymers, ASTM D3165 was employed as the basis for measuring the tensile shear strength of the Cu foil specimens glued with BCC. The results from the performance tests are shown in Figure 6.

In sharp contrast to the evolution in mechanical properties of the cured BCC film, the tensile shear strength of the Cu foil bonded specimens initially increased and then decreased as SS loading rose from 0% to 60%. When the weight ratio of SS against BCC film was 50%, it endowed the adhesives with the highest average strength. The maximum shear stress for 50% SS-SA and 50% SS-CMC/SBR were 1.89 and 0.71 MPa, respectively, which were separately eight times and twice of that for unfilled polymers. The results demonstrated that an appropriate amount of SS had a fairly positive influence on the bonding performance of the electrode adhesive system. In addition, the neat SA and 10% SS-SA provided lower shear strength than CMC/SBR. However, SA-based composites with relative high SS loading (>15%) had much better adhesion properties than CMC/SBR-based counterparts at the same content of carbon black; this difference further enlarged as the SS concentration increased from 20% to 50%. Remarkably, SA adhesives with 50% SS offered at least twice the resistance to shear failure than 50% SS-CMC/SBR. From a mechanical point of view, these results may well explain why the electrodes with the SA binder exhibited much better electrochemical cyclic performance than the counterparts bonded with CMC/SBR.

**Figure 7:**
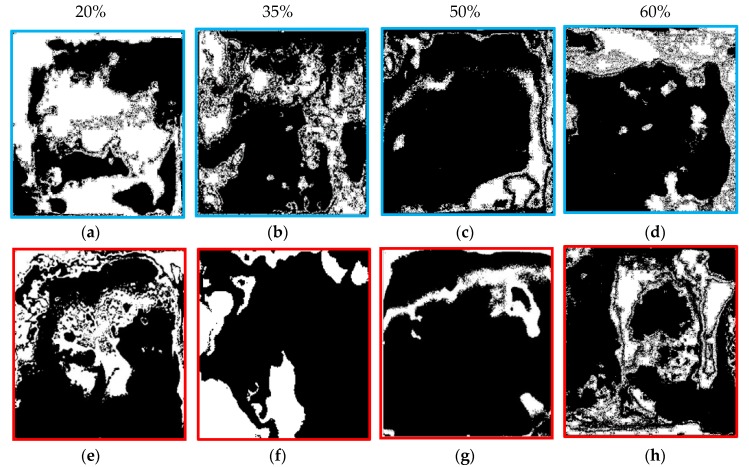
Optical photographs of failure interface between current collector (white color) and BCC (black color) containing various carbon black contents (CMC/SBR: (**a**–**d**); SA: (**e**–**h**)).

**Figure 8:**
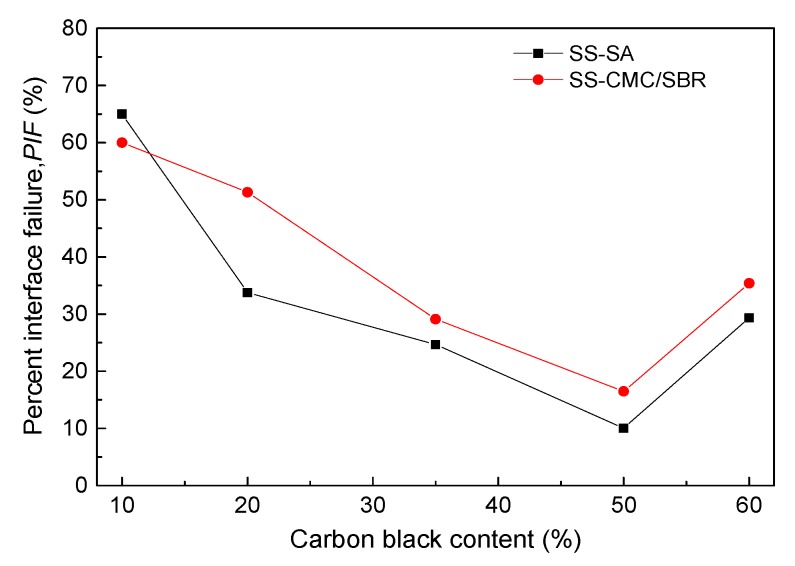
Effect of SS content on percent interface failure (PIF) of BCC with various aqueous polymers.

In order to go deep into understanding the effect of SS loading on the bonding performance of SA and CMC/SBR, an optical microscope was employed to check the surface morphology of the rupture interface between BCC binder and Cu foil. As shown in Figure 7, the surface of Cu foil after the tensile shear test can be divided into white and black regions, which represent the Cu current collector and BCC coating, respectively. It is clearly observed that the black zone in Cu surface gradually enlarged as carbon black concentration in BCC increased from 20% to 50%, which indicated that predominant fracture modes increasingly switched from “adhesion failure” to “cohesion failure”. In another word, bonding strength was primarily dependent of the mechanical properties of BCC coating. On the basis of the strength theory, an increase in the ratio of strength to modulus (σ*_b_*/*E*) boosted the mechanical integrity. As higher SS content (0–50%) in the composite lead to enlargement of σ*_b_*/*E*, as seen in Figure 2e, it can be understood that BCC doped with SS loading of 50% offered the best results for bonding the Cu current collector. However, excess carbon black particles also reduced the resistance of BCC-bonded Cu to the interface rupture due to the decrease in ratio of strength to stiffness of the BCC coating layer. Moreover, compared with the surface morphology of the failure interface between SS-CMC/SBR and Cu, much of current collector surface was covered with SS-SA coating at the same content of carbon black. In terms of the area ratio of white region to whole cross-section, percent interface failure (PIF) against SS loading was obtained as plotted in Figure 8.

As shown in Figure 8, by increasing the carbon black to 50% loading of composite adhesives, the percent interface failure of Cu specimens glued by BCC significantly decreased to less than 20%. Nevertheless, at a higher weight ratio of SS (≥60%), the value of PIF rapidly rose regardless of the SA or CMC/SBR matrices. Further, “adhesion failure” was again controlled by the rupture process for the polymer adhesive with 60% SS. This finding was consistent with many reported results of electrochemical stability for secondary battery electrodes that consisted of conductive agent and polymer adhesive with a weight ratio of 1:1. As expected, SA-based composites showed higher bonding strength than the CMC/SBR system at the normal contents (≥20%) of carbon black in BCC recipe for secondary battery electrode.

## 4. Conclusions

(1) The ratio of tensile strength to stiffness and rupture elongation of the cured composite films rose when Super-S carbon black (SS) was increased in addition to the aqueous polymer. Meanwhile, the characteristic relaxation time markedly decreased.

(2) The tensile shear strength of the composite adhesives with 50% SS greatly augmented to at least twice of that for neat polymers. “Cohesion failure” of coating dominantly controlled the rupture mechanism. Suitable loadings of SS greatly reduced the percent interface failure of the aqueous polymer-bonded Cu current collector. However, excess carbon black loading did not maintain its enhancing effect and resulted in a weakening adhesive layer.

(3) At the same content of conductive agent, SS-SA exhibited much better tensile properties and bonding performance when compared to SS-CMC/SBR. Its rate of relaxation was also higher than its counterparts.

(4) When carbon black particles were doped into the aqueous polymer at the weight ratio of 1:1, it not only endowed the highest the ratio of strength to stiffness of cured film but also had adhesive properties regardless of SA or CMC/SBR.
